# The microRNA-15a-PAI-2 axis in cholangiocarcinoma-associated fibroblasts promotes migration of cancer cells

**DOI:** 10.1186/s12943-018-0760-x

**Published:** 2018-01-18

**Authors:** Penkhae Utaijaratrasmi, Kulthida Vaeteewoottacharn, Takaaki Tsunematsu, Pranisa Jamjantra, Sopit Wongkham, Chawalit Pairojkul, Narong Khuntikeo, Naozumi Ishimaru, Yongyut Sirivatanauksorn, Ananya Pongpaibul, Peti Thuwajit, Chanitra Thuwajit, Yasusei Kudo

**Affiliations:** 1 Graduate Program in Immunology, Department of Immunology, Faculty of Medicine Siriraj Hospital, Mahidol University, Bangkok, Thailand; 2 Department of Biochemistry, Faculty of Medicine, Khon Kaen University, Khon Kaen, Thailand; 3 Department of Pathology, Faculty of Medicine, Khon Kaen University, Khon Kaen, Thailand; 4 Department of Surgery, Faculty of Medicine, Khon Kaen University, Khon Kaen, Thailand; 5 Cholangiocarcinoma Research Institute, Khon Kaen University, Khon Kaen, Thailand; 6 Department of Oral Molecular Pathology, Tokushima University Graduate School of Biomedical Sciences, Tokushima, Japan; 7 Department of Surgery, Faculty of Medicine Siriraj Hospital, Mahidol University, Bangkok, Thailand; 8 Department of Pathology, Faculty of Medicine Siriraj Hospital, Mahidol University, Bangkok, Thailand; 9 Department of Immunology, Faculty of Medicine Siriraj Hospital, Mahidol University, Bangkok, Thailand

**Keywords:** Cancer-associated fibroblasts, microRNA (miRNA), Cholangiocarcinoma, PAI-2, Migration, Tumor microenvironment

## Abstract

**Background:**

Cholangiocarcinoma (CCA) has an abundance of tumor stroma which plays an important role in cancer progression via tumor-promoting signals. This study aims to explore the microRNA (miRNA) profile of CCA-associated fibroblasts (CCFs) and the roles of any identified miRNAs in CCA progression.

**Methods:**

miRNA expression profiles of CCFs and normal skin fibroblasts were compared by microarray. Identified downregulated miRNAs and their target genes were confirmed by real-time PCR. Their binding was confirmed by a luciferase reporter assay. The effects of conditioned-media (CM) of miRNA mimic- and antagonist-transfected CCFs were tested in CCA migration in wound healing assays. Finally, the levels of miRNA and their target genes were examined by real-time PCR and immunohistochemistry in clinical CCA samples.

**Results:**

miR-15a was identified as a downregulated miRNA in CCFs. Moreover, *PAI-2* was identified as a novel target gene of miR-15a. Recombinant PAI-2 promoted migration of CCA cells. Moreover, CM from miR-15a mimic-transfected CCFs suppressed migration of CCA cells. Lower expression of miR-15a and higher expression of PAI-2 were observed in human CCA samples compared with normal liver tissues. Importantly, PAI-2 expression correlated with poor prognosis in CCA patients.

**Conclusions:**

These findings highlight the miR-15a/PAI-2 axis as a potential therapeutic target in CCA patients.

**Electronic supplementary material:**

The online version of this article (10.1186/s12943-018-0760-x) contains supplementary material, which is available to authorized users.

## Background

Cholangiocarcinoma (CCA) is a tumor of bile duct epithelium which arises from cholangiocytes or the epithelial cells lining the biliary tree [1]. It is a major health problem in the endemic area of *Opisthorchis viverrini* infection in Southeast Asia especially in the northeastern part of Thailand [2]. It is well known that CCA predominantly shows an abundant desmoplastic reaction in the tumor stroma [3]. These alpha-smooth muscle actin (ASMA) positive stromal cells were correlated with the degree of tumor fibrosis in CCA tissues [4] and levels were significantly related to poor prognosis of CCA patients [5].

Cancer-associated fibroblasts (CAFs) are the major component in the stromal environment of several carcinomas including CCA and mainly affect tumor progression through the induction of cancer cell growth, invasion, and metastasis [6, 7]. The current authors have previously reported that CAFs in CCA tissues have a high expression of ASMA and the level of ASMA-positive CAFs was correlated with poor patient survival [5]. In addition, the conditioned-media (CM) from CAFs induced CCA cell proliferation by driving cancer cells into the G2/M phase of the cycle. Previous exploration of the gene expression profile of CAFs isolated from CCA tissues found a set of genes which encoded secreted tumorigenic protein products that could act in a paracrine manner to promote cancer progression [8].

MicroRNAs (miRNAs) are small, non-coding single-stranded RNAs and one of the key post-transcriptional gene expression regulators [9, 10]. They act as negative regulators to control gene expression via imperfect or perfect binding to their target mRNAs. miRNA regulation in CAFs has been studied in several cancers [11–17]. Microarray analyses identified specific expression of several miRNAs in CAF within urinary bladder cancer and breast cancer compared with fibroblasts from non-cancer tissues [12, 16]. The impact of miRNAs underlying gene expression control in CCA-associated fibroblasts (CCFs) has not so far been reported. This study aimed to explore the miRNA profiles of CCFs by miRNA microarrays and to identify the aberrant target mRNAs; in particular, those that encode secreted tumorigenic proteins.

## Methods

### Cell culture

Human CCA cell lines, KKU-213 and KKU-055 were cultured in Hams F-12 Nutrient Mixture (Invitrogen) supplemented with 10% heat-inactivated fetal bovine serum (FBS). Antibiotics including 0.1 U/ml penicillin G sodium and 0.1 mg/ml streptomycin (Gibco BRL), and 5 mg/ml amphotericin B (Amphotret™, Bharat Serums and Vaccines Limited Ltd.) were added to prevent microbial contaminations. CCFs were isolated from CCA tissues obtained from patients who underwent hepatectomy at Srinakarind Hospital. The protocol was approved by the Khon Kaen University Ethics Committee for Human Research (HE521209 and HE571283). Each patient was informed about tissue collection and signed the consent form. The pieces of tissue were submerged in antibiotic-containing Dulbecco’s Modified Eagle Media (DMEM; Gibco BRL) with 10% FBS for 30-60 min to reduce microbial contamination during the process of sample collection and transfer. The samples were rinsed with 1X phosphate buffer saline (PBS) and cut into small pieces (2 mm^3^). Fresh complete medium, 10% FBS-containing DMEM with 20 mM HEPES (Gibco BRL), was used to wash out cell debris. Pieces of tissue were transferred to fresh culture vessels and allowed to adhere at 37 °C in a 5% CO_2_ in air incubator. After fibroblast-like cells emerged from beneath the tissue pieces, the adherent cells were trypsinized with 0.25% trypsin/EDTA (Gibco BRL). The complete medium was replaced every 3 days. Cells were subcultured for 7 to 23 passages and used for the reported studies. Normal skin fibroblasts (SFs) were obtained from human tissues and cultured in a similar way.

### Immunocytochemistry

To identify primary cultures of fibroblasts from contaminating cancer cells, the absence of epithelial markers and the presence of fibroblast-related mesenchymal markers were checked. Cytokeratin 19 (CK19), an epithelial marker; vimentin (VIM) and alpha-smooth muscle actin (ASMA), mesenchymal markers, were selected. CCA cell lines were used as the positive controls for CK19 detection. Cells were cultured for 24 h in 96-well plates and fixed with 4% paraformaldehyde for 15 min and permeabilized with 1% Triton X-100 for 1 min. Non-specific binding was blocked by 1% BSA in 1X PBS for ASMA and 5% FBS in 1X PBS for VIM and CK19. The mouse anti-human CK19 antibody (1:100 dilution; SC-6278, Santa Cruz Biotechnologies Inc.), mouse anti-human VIM antibody (1:500 dilution; SC-6260, Santa Cruz Biotechnologies Inc.), and mouse anti-human ASMA antibody (1:200 dilution; Sigma-Aldrich) were used. The primary antibody was incubated for 3 h at room temperature. After washing, goat anti-mouse IgG-Cy3 (Jackson Immunoresearch Laboratories Inc.) was added for 1 h at room temperature followed by adding Hoechst stain (Invitrogen). The signals were detected under an inverted fluorescence microscope.

### Fibroblast conditioned-media collection

CM from CCFs and SFs was collected by growing cells in complete medium for 4 days until cells reached about 90% confluency. Then, cells were washed twice with PBS and incubated in DMEM with 2% FBS for 48 h. After that, CM was collected, centrifuged at 4 °C 1000 g for 5 min to remove cell debris, sterile filtered and stored at -80 °C until use.

### Wound healing migration assay

CCA cells were cultured in 6-well plates until they reached approximately 90% confluence. A reference line was drawn in the center of the underside of the plate. The cell monolayer was scratched using a sterile 1000-μl pipette tip followed by washing three times with fibroblast CM, serum-free medium or complete medium. The scratched areas indicated by reference lines were recorded at the beginning and 24 h after treatment. The migration efficiency was expressed as the percentage closure of the wound area calculated by the following formula:

$$ \%\mathrm{wound}\  \mathrm{healing}=\kern0.5em \frac{\mathrm{wound}\  \mathrm{space}\ \mathrm{at}\ 0\ \mathrm{h}-\mathrm{wound}\  \mathrm{space}\ \mathrm{at}\ 24\ \mathrm{h}}{\mathrm{wound}\  \mathrm{space}\ \mathrm{at}\ 0\ \mathrm{h}}\times 100 $$

### Cell proliferation assay

Cells were suspended with 5000 cells/well in 96-well plates for 24 h. Culture medium was then changed to 100 μl/well of 10% FBS in DEMEM or CM and the plate was incubated for 24 h and 48 h in a 5% CO_2_ incubator at 37 °C. Cell viability was measured by MTS assay (Promega, Mandison, WI) following manufacturer’s instruction. The absorbance was detected at 490 nm with a microplate reader. The assays were performed in duplicate.

For checking the effect of recombinant target proteins, 8000 cells/well were suspended for 24 h. Then, culture medium was changed to fresh medium with or without 2 μg/ml of human recombinant WNT10B protein or 2 μg/ml of human recombinant PAI-2 protein in 96-well plates and the plates were incubated for 24 h. Finally, proliferating cell numbers were determined using the cell-counting kit 8 (CCK-8) solution (Dojindo Molecular Technique, Inc.). 10 μl of CCK-8 solution was added to each well and incubated for 1 h in a 5% CO_2_ incubator at 37 °C. The number of proliferating cells was determined by absorbance measured at 450 nm using a microplate reader at 0, 24 and 48 h.

### miRNA microarray analysis

miRNA expression profiles of CCFs and SFs were analyzed by the TaqMan® Array Human MicroRNA Card A or Card B Set v2.0 (Applied Biosystems). Total RNA was extracted by MirVana® microRNA isolation kit (Applied Biosystems). Then 480 ng of total RNA was converted to cDNA by Megaplex™ RT stem-loop primer pools A and Megaplex™ RT stem-loop primer pools B (Applied Biosystems) following the instruction manual. RT reaction was performed using Megaplex™ RT primers, human pools A, B and TaqMan® MicroRNA Reverse Transcription Kit (Applied Biosystems). cDNAs obtained were amplified with TaqMan® Human MicroRNA Array A, B and TaqMan® Universal Master Mix II, no UNG using Real time PCR ABI7900HT (Applied Biosystems). The amplification reactions were monitored using SDS software v2.3, and data analyzed with RQ Manger 1.2. The fold changes of each CCF sample were compared with average expression of SFs after normalization with U6 small nuclear RNA (snRNA). Fold changes >2-fold were taken to indicate up-regulated miRNAs and ≤0.5-fold down-regulated miRNAs.

### Real-time PCR

To evaluate miRNAs, total RNA was isolated from cells using the MirVana® microRNA isolation kit (Applied Biosystems). The isolates were quantified and their purity was evaluated using a spectrophotometer. The cDNA was synthesized from 10 ng of total RNA using a TaqMan® MicroRNA Assay and a TaqMan® MicroRNA Reverse Transcription Kit (Applied Biosystems). Synthesized cDNA was amplified with a TaqMan® MicroRNA Assay and a TaqMan® Universal Master Mix II, no UNG (Applied Biosystems) using an ABI 7300 real time PCR system (Applied Biosystems) for 45 cycles of denaturation at 95 °C for 15 s, and annealing and extension at 60 °C for 60 s. Data were normalized with U6 and each analysis was performed three times. The average of three trials was used for statistical analysis. ANOVA was used for comparison of variables in more than two groups and a Student’s T-test was used for comparisons between two groups. A *P*-value <0.05 was considered statistically significant.

To evaluate mRNAs, 1 μg of total RNA was converted to cDNA by ReverTra Ace-α® (Toyobo Co; Ltd) and 50 ng of cDNA was amplified with SYBR® Premix Ex Taq™ II (Takara Bio Inc.). The reaction was performed using a DNA Engine Opticon Real-Time Thermal Cycler (MJ Research, Inc.) under the following conditions; heat-denaturation at 95 °C for 10 min followed by cycled amplification at 95 °C for 15 s and 60 °C for 45 s for 50 cycles. The primer sequences are shown in Additional file 1: Table S1 and Additional file 2: Table S2. Data were normalized with GAPDH and each analysis was performed three times. The average of three trials was used for statistical analysis. The ANOVA test was used for comparison of variables in more than two groups and the Students T-test was used for two groups. A *P*-value <0.05 was considered statistically significant.

### miRNA target gene prediction

The potential target genes of miR-15a and miR-148a were determined using TargetScanHuman 6.2 (http://targetscan.org/). To further reduce the false target genes in the analysis, target genes that were co-predicted target genes by at least two of the three data sets were considered as potential targets. According to these algorithms, protein array chip data and mRNA microarray chip data from a previous report [8] were used for screening the most suitable candidate target genes. Candidate targeted genes which encoded the secreted proteins, were selected as targeted genes from overlapping at least 2 of 3 algorithms or TargetScan and have been found in a previous report [8] and/or previous reports from PubMed data.

### Transfection of miRNA mimic and miRNA inhibitors

The hsa-miRNAs mimics were commercially available (Ambion®, Thermo Scientific). The product numbers MH10235 and MH1026 were used as a miR-15a and miR148a mimics. The negative control miRNA mimic (mirVana™ miRNA mimic negative control #1) was used as the scrambled miRNA mimic. The anti-mir-15a and miR-148a inhibitors, MC10235 and MC10263, were obtained from Ambion®, Thermo Scientific, whereas a negative control inhibitor, mirVana™ miRNA inhibitor negative control #1, were used as the scrambled miRNA inhibitor. Cells (CCFs) were transfected with 5 nM hsa-miR-15a, hsa-miR-148a and used as the negative control miRNA mimic using Lipofectamine RNAi Max (Invitrogen) according to the manufacturer’s protocol. Cells (SFs) were transfected with 75 nM of miR-15a inhibitor, miR-148a inhibitor and negative control miRNA inhibitor using Lipofectamine RNAi Max. Efficiency of miRNA inhibition or mimic transfection was confirmed at the mRNA level by real-time PCR.

### Luciferase reporter assay

The full-length 3′-UTR of human VEGFA, PAI-2, and WNT10B was amplified by PCR from genomic DNA and cloned at the EcoRI site into pmirGLO vector (Promega). For PAI-2, we also generated a deletion mutant of putative miR-15a binding sequences, TGCTGCT, from full-length 3′-UTR of human PAI-2 construct. We used a deletion mutant of pmirGLO-PAI-2 as a negative control. This construct was confirmed by sequencing. For luciferase activity analysis, this vector was co-transfected with miRNA duplex in 293 T cells using DharmaFECT Duo transfection reagent (Thermo Fisher Scientific) for 72 h, and luciferase assays were performed with the Dual-Luciferase reporter system (Promega) according to the manufacturer’s instructions. Luminescent signal was quantified by a luminometer (Glomax; Promega), and each value from the firefly luciferase construct was normalized against the Renilla luciferase assay.

### Double chamber migration assay

24-well cell culture inserts with 8 μM pores (3097, Falcon, Becton Dickinson) were used. After trypsinization, 1.5 × 10^5^ CCA cells were resuspended in CM under different conditions and placed in the upper compartments of the cell culture inserts for 7 h. In an additional assay, 2 μg/ml of human recombinant WNT10B protein (7196-WN-010, R&D systems) or 2 μg/ml of human recombinant PAI-2 protein (P6129, Abnova) was added after trypsinization and before plating the cells on the upper compartments of the inserts. After incubation at 37 °C, the cells that migrated through the membrane to the lower side were fixed with formalin and then stained by hematoxylin. The number of migrated cells were determined by counting the cells on the lower side of the filter under a microscope at ×100 magnification. The filters were assayed 3 times and 3 fields were randomly selected and counted for each assay.

### Western blot analysis

Sample protein concentrations were measured by the Coomassie Plus™ (Bradford) Assay Kit (Thermo scientific), and 30 μg total protein/lane were subjected to electrophoresis on 10% polyacrylamide gels followed by electroblotting onto polyvinylidene fluoride (PVDF) membranes. The membranes were blocked with 3% milk in TBS-T and incubated overnight at 4 °C with anti-human PAI-2 polyclonal antibody (Santa Cruz). The membranes were then washed with TBS-T and incubated with HRP-conjugated goat anti-rabbit IgG antibody (ab6721, Abcam, Cambridge, MA, USA) and the proteins visualized using SuperSignal West Pico Chemi-luminescent Substrate under Gel Document Synene (Syngene). β-actin was used as a loading control. For CM, PAI-2 level was quantitated by densitometric analysis of the protein bands against that of the total protein loading using ImageJ software (National Institutes of Health, Bethesda, MD, USA).

### Immunohistochemistry in CCA tissues

Seventy-four cases of intrahepatic CCA were used in this study. All patients were preoperatively chemo- and radiotherapy naïve. All tissue samples with their clinicohistopathological records were obtained from the Department of Pathology, Faculty of Medicine Siriraj Hospital, Mahidol University Faculty of Medicine and Departments of Biochemistry, Pathology, and Surgery Faculty of Medicine, Khon Kaen University under the approval of the Ethics Committee for Human Research (HE521209 and HE571283) and Siriraj Institutional Review Board (si287/2011). The demographic data of all the patients in this study are summarized (Table 1).

**Table 1:** Univariate analysis and Cox regression multivariate analysis of PAI-2 in cancer cells in clinical CCA samples and patient clinicopathological parameters

Parameters (no. of cases)		Univariate			Multivariate	
		PAI-2 in cancer cells		*P*-value	OR (95% CI)	*P*-value
		High	Low			
High PAI-2 expression (72)		45	27	–	3.452 (1.188-10.030)	0.023
Age (y) (72)	< 60	24	13	0.808	1.00 (0.499-2.003)	1.000
	≥ 60	21	14			
Gender (72)	Male	25	17	0.625	1.291 (0.683-2.443)	0.432
	Female	20	10			
Tumor size (cm) (67)	< 5	15	9	0.790	1.375 (0.698-2.705)	0.357
	≥ 5	29	14			
Tumor staging (72)	I - III	8	10	0.093	0.963 (0.359-2.579)	0.940
	IV	37	17			
Histological type (72)	WD	23	9	0.220	–	0.008
	MD	6	4	1.000	2.034 (0.915-4.519)	0.081
	PD	2	5	0.095	4.414 (1.1610-12.106)	0.004
	Pap	14	9	1.000	6.199 (1.754-21.909)	0.005
Vascular invasion (51)	Absence	18	16	0.065	0.405 (0.190-0.866)	0.020
	Presence	14	3			
LN metastasis (72)	Absence	28	20	0.439	2.078 (0.925-4.670)	0.077
	Presence	17	7			

*WD* well differentiated, *MD* moderately differentiated, *PD* poorly differentiated, *Pap* papillary, *OR* odd ratio, *95% CI* 95% confidence interval, *LN* lymph node

Tissue sections were deparaffinized in xylene, rehydrated in descending grades of ethanol. Heat-induced antigen retrieval was performed in a 95 °C water bath for 1 h by incubation with 0.05 M EDTA buffer pH 8.0. Endogenous peroxidase activity was blocked with methanol containing 0.3% H_2_O_2_ for 30 min followed by blocking non-specific binding sites with 3% bovine serum albumin in 1× PBS for 1 h at room temperature. The sections were treated with polyclonal human PAI-2 antibody (Santa Cruz, 1:100) at 4 °C overnight. The EnVision^+^ System-HRP labelled polymer anti-rabbit (K4003, Dako, Carpinteria, CA, USA) secondary antibody was used. The immunoreactive signal was developed by Liquid DAB^+^ Substrate Chromogen System (K3467, Dako) and counterstained with hematoxylin. Tissue slides were dehydrated, mounted, and further analyzed using a ScanScope® scanner. Sections were semi-quantitatively evaluated and scored by two pathologists; both were blinded to the clinical parameters. For grading the immunohistochemical results, the staining intensities were recorded separately for cancer cells and CCFs. The I scale of intensity: 0 (negative), 1 (weak), 2 (moderate), and 3 (strong) was used whereas the percentage of positive cells (P) in the area was scored as: 0, 1 (1-25%), 2 (26-50%), 3 (51-75%) and 4 (>75%). The multiplication of P and I resulted in the total score of 0-12. The PAI-2 immunohistochemical score of more than 6 was classified as the high expression group, whereas those with ≤ 6 total score was graded as the low expression group.

### Statistical analyses

The differences between the test and the control conditions in this study were statistically analyzed from the mean ± SD of three independent experiments. Chi-square or Fisher’s exact tests were used to analyze the clinicopathohistological relevance of PAI-2 in clinical CCA samples. The Kaplan-Meier method and Log-Rank tests were used for survival analysis. Univariate and multivariate Cox regression analyses of potential factors were performed to identify the significant predictors. All the statistical analyses were performed using SPSS 17.0 statistical software. *P*-values of less than 0.05 were considered to be statistically significant.

## Results

### Characterization of CCFs isolated from CCA tissues

**Fig. 1:**
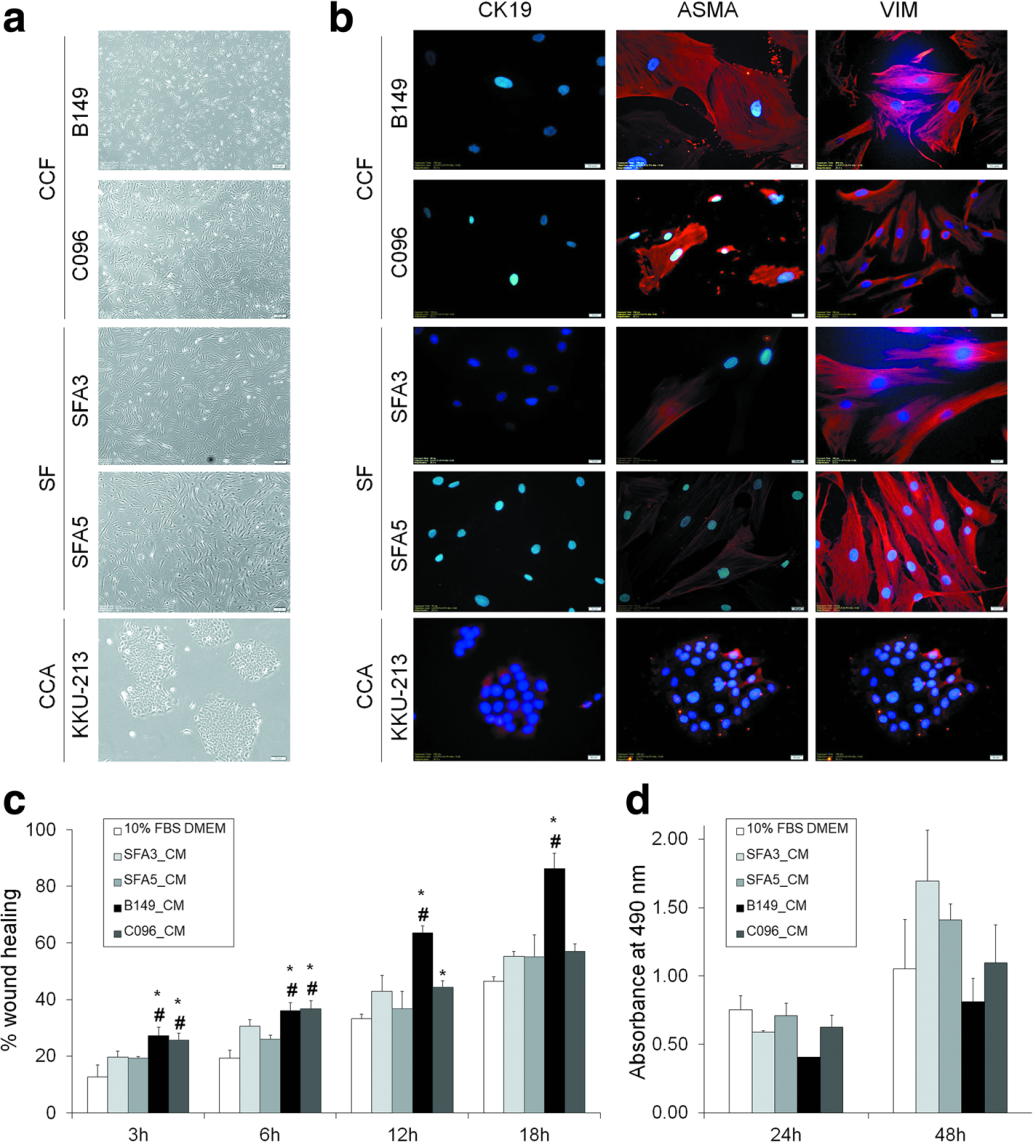
Characterization of CCFs. **a** Morphology of CCFs, SFs and CCA cells. Original magnification of 40×. **b** Immunocytochemical staining of CK19, VIM and ASMA. The KKU-213 CCA cell line was used as a positive control of CK1 expression. Original magnification of 400×. **c** The effect of CCFs-CMs and SFs-CMs on migration of CCA cells in a wound healing assay. At 3, 6, 12, and 18 h, the % of wound area was measured. Bars represent mean ± SD of three measurements. *P < 0.05 (compared to 10% FBS DMEM). ^#^*P* < 0.05 (compared to SF_CM). **d** The effect of CCFs-CMs and SFs-CMs on proliferation of CCA cells. Bars represent mean ± SD of two measurements

The CCFs (B149 and C096) and normal control SFs (SFA3 and SFA5) showed spindle-like shapes in contrast to the cobble-stoned shape of KKU-213 CCA cells (Fig. 1a). In CCFs and SFs, immunocytochemical analysis revealed negative staining of the epithelial marker CK19 and positive staining of VIM (Fig. 1b). CCFs showed higher expression of ASMA than SFs (Fig. 1b). The CM from both CCFs revealed a significantly greater ability to induce migration in CCA cells than normal SFA3 and SFA5 cells at 3 and 6 h (Fig. 1c). CM from CCFs showed no effect on proliferation of CCA cells (Fig. 1d). The expression of CCF-related genes that were previously reported [8], included a disintegrin and matrix metalloproteinase 12 (*ADAM12*)*,* amphiregulin (*AREG*), epiregulin (*ER*)*,* jagged 1 (*JAGL1*)*,* platelet-derived growth factor-alpha (*PDGFA*), secretogranin 2 (*SCG2*), periostin (*PN*), fibroblast activation protein (*FAP*), fibroblast specific protein (*FSP*), *ASMA*, and *VIM* (Additional file 3: Figure S1). *ASMA* and *PDGFA* expression levels were higher in CCFs than in SFs (Additional file 3: Figure S1).

### Comparing miRNA expression profiles between CCFs and SFs

**Fig. 2:**
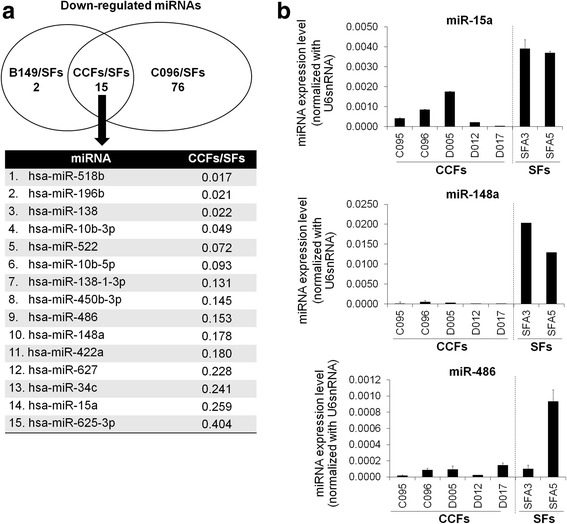
Comparing miRNA expression profiles between CCFs and SFs. **a** Vane diagram and lists of down-regulated miRNAs showing the fold change in CCFs versus SFs. **b** Real-time PCR of miR-15a, miR-148a and miR-486 expression levels in 5 CCFs and 2 SFs. Bars represent mean ± SD of three measurements

The PCR-based miRNA array was performed to measure the miRNA profiling in 2 CCFs compared to 2 SFs. Among 677 human miRNAs, 162 miRNAs were up-regulated (≥ 2-fold) and 93 miRNAs were downregulated (≤ 0.5-fold) in both CCFs (B149 and C096) compared to the expression of either SFA3 or SFA5 cells. The downregulated miRNAs comparing B149 cells to SFs (B149/SFs) and C096 cells to SFs (C096/SFs) were 17 and 91 miRNAs (Fig. 2a) respectively. Among them, 15 miRNAs (CCFs/SFs) were commonly downregulated in CCFs compared to both SFs after normalization with U6 snRNA (Fig. 2a and Additional file 4: Figure S2). From a literature review, among 15 miRNAs, miR-15a, miR-148a and miR-486 targeted several secreted proteins (Additional file 5: Table S3). The expression levels of these miRNAs in CCFs and SFs were examined and only miR-15a and miR-148a showed decreased levels in all CCFs in comparison to those in SFs (Fig. 2b). Moreover, B149/SFs and C096/SFs revealed 145 and 4 up-regulated miRNAs, with 13 miRNAs in common after normalization with U6 snRNA (Additional file 6: Figure S3A and B). In this study, we focused on downregulated miRNAs including miR-15a and miR-148a.

### Identification of miR-15a target genes in CCFs

**Fig. 3:**
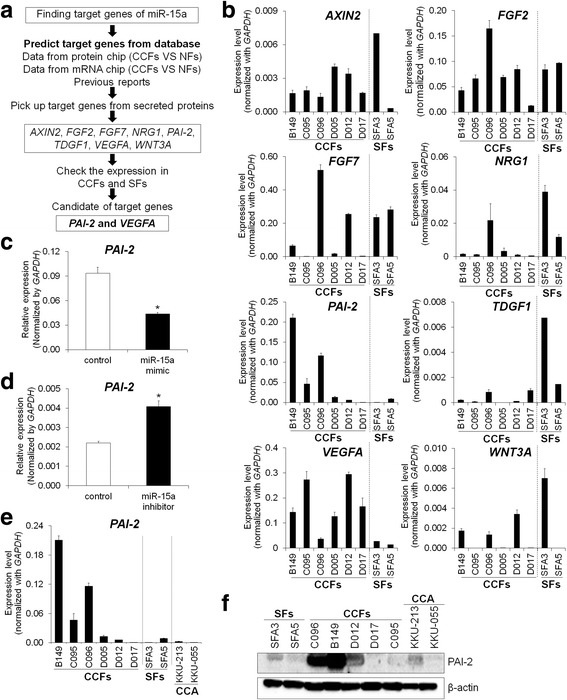
Identification of miR-15a target genes. **a** Four criteria for finding the candidate target genes of miR-15a. **b** The expression levels of eight predicted target genes of miR-15a in 6 CCFs compared to 2 SFs. Bars represent mean ± SD of three measurements. **c** Expression of *PAI-2* in miR-15a mimic-transfected C096 cells by real-time PCR. Scrambled miRNAs were used as negative control miRNA. **d** Expression of *PAI-2* in miR-15a inhibitor transfected SFs. *P < 0.05 compared to control. **e** Expression of *PAI-2* in 6 CCFs, 2 SFs and 2 CCA cell lines. Bars represent means ± SD of three measurements. **f** PAI-2 expression was examined by Western blot analysis in 2 SFs, 5 CCFs, and 2 CCAs. β-actin was used as a loading control

To predict the potential mRNA targets of miR-15a and miR-148a, TargetScan miRNA target prediction database was used. Taken together, these lists of target genes with the overexpressed genes in CCFs studied by cDNA microarray [8] and highly expressed proteins in the CM (unpublished data), propose several target genes of interest for miR-15a including *VEGFA, PAPPA, NRG1, FGF2, PAI-2, AXIN2, FGF7*, and *WNT3A* (Fig. 3a) and for miR-148a are *TNFRSF6B, CD62L* (*L-selectin*)*, TGFA, WNT1,* and *WNT10B* (Additional file 7: Figure S4A). The real-time PCR results revealed that only *VEGFA* and *PAI-2* were up-regulated in CCFs, in comparison with SFs (Fig. 3b). *WNT10B* was also up-regulated in CCFs, compared with SFs (Additional file 7: Figure S4B).

It has already been shown that *WNT10B* is a target of miR-148a in CAFs [17]. We confirmed that *WNT10B* expression was downregulated by ectopic overexpression of miR-148a and was upregulated by the miR-148a inhibitor (Additional file 7: Figure S4C and D). In this study, the focus was on *PAI-2* and *VEGFA* as targets of miR-15a. The expression levels of *PAI-2* and *VEGFA* after transfection of miR-15a mimic in CCFs were examined. Reduced expressions of *PAI-2* and *VEGFA* were observed by miR-15a mimic-transfection compared to control miRNA transfection (Fig. 3c and Additional file 8: Figure S5A). Moreover, we confirmed that the miR-15a inhibitor increased the expression of *VEGFA* and *PAI-2* in SFs (Fig. 3d and Additional file 8: Figure S5B).

The mRNA expression levels of these two target genes of miR-15a were checked in CCFs, SFs and CCA cell lines (KKU-213 and KKU-055). Expressions of *PAI-2* and *VEGFA* in CCFs were higher than those in SFs (Fig. 3e and Additional file 8: Figure S5C). Interestingly, expression of *PAI-2* in CCF was higher than that in CCA cells (Fig. 3e and f), suggesting that CCFs may be a major source of PAI-2 protein, although cancer cells could also produce PAI-2. In the following experiments, we focused on the miR-15a-PAI-2 axis.

### miR-15a-PAI-2 axis in CCFs promotes migration of CCA

**Fig. 4:**
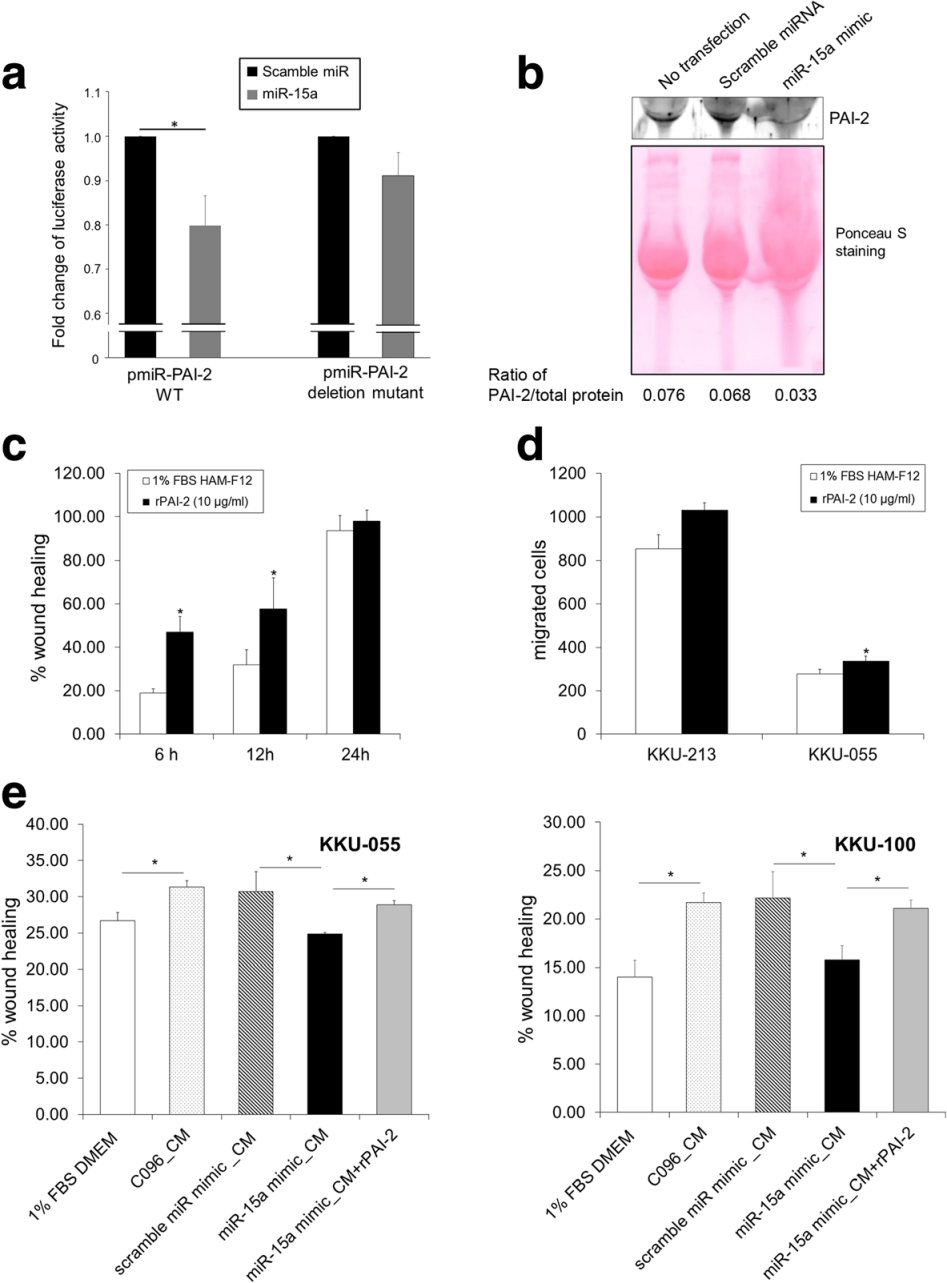
The miR-15a-PAI-2 axis promotes migration of CCA cells. **a** Luciferase assay of pmirGLO vector containing wild type 3’-UTR of *PAI-2* and its deletion mutant of putative miR-15a binding sequences in mature miR-15a-transfected cells. The data were normalized to scrambled miRNA transfected cells. Bars represent mean ± SD of five measurements. **b** Secreted PAI-2 in CM from miR-15a mimic-transfected C096 CCFs examined by Western blot analysis. CMs from cells with no transfection and scrambled miRNA-transfected cells were used as a control. Ponceau S staining of each sample on the membrane was shown. Densitometric analysis of PAI-2 normalized against the total protein loading is shown. **c** The effect of rPAI-2 on migration of KKU-213 CCA cells was examined by a wound healing assay at different time points. Graphs show % of wound areas in KKU-213 cells with or without rPAI-2 in. Bars represent mean ± SD of three measurements. **d** The effect rPAI-2 on CCA cells in the chamber migration assay. Graphs show the number of migrated cells with or without rPAI-2 treatment at 7 h. Bars represent mean ± SD of three measurements. **e** The effect of CM from miR-15a mimic or scrambled miRNA transfected C096 CCFs on the migration of CCA cells with or without rPAI-2. The migration was evaluated at 12 h by a wound healing assay as previously described. Bars represent mean ± SD of three measurements. *P < 0.05

To investigate whether miR-15a can directly target PAI-2 by interacting with its 3′-UTR in vitro, we amplified the full-length of 3’-UTR from genomic DNA. The putative miR-15a binding sequences are TGCTGCT. We deleted a deletion mutant of putative miR-15a binding sequences from full-length 3′-UTR of human PAI-2 construct and used as a negative control. The wild type and mutant 3′-UTR of PAI-2 were cloned and inserted downstream of a luciferase reporter gene. Subsequently, miR-15a mimic or control miRNA was co-transfected with these reporter vectors into 293 T cells. The miR-15a mimic significantly decreased the relative luciferase activity of the wild type 3′-UTR reporter vector, but not statistical significance for the deletion mutant (Fig. 4a).

As PAI-2 is a secreted protein, we confirmed the expression of PAI-2 in CM from C096 cells (Fig. 4b). To examine the effects of PAI-2 on the proliferation and migration of CCA cells, we used rPAI-2 protein. The rPAI-2 had the capability to increase migration during 6-12 h of exposure compared with untreated controls with a statistical significance in CCA cells (Fig. 4c and d). Notably, rPAI-2 treatment did not promote proliferation (Additional file 9: Figure S6A) or invasion (Additional file 9: Figure S6B, see method in Additional file 10). The level of miR-15a in CCFs was increased after transfection without any cytotoxic effect (data not shown). Next, we examined the effects of CM from miR-15a mimic-transfected C096 CCFs on the migration of KKU-055 and KKU-100 CCA cells by the wound healing assay. Ectopic overexpression of miR-15a mimic in C096 cells downregulated PAI-2 expression in CM (Fig. 4b). Moreover, the CM from miR-15a mimic-transfected C096 CCFs significantly inhibited the migration of CCA cells in comparison with that from scrambled miRNA transfected cells (Fig. 4e). Interestingly, rPAI-2 treatment partially rescued the inhibitory effect of migration by CM from miR-15a mimic-transfected CCFs in both CCA cell lines (Fig. 4e).

### PAI-2 expression in clinical CCA tissues

**Fig. 5:**
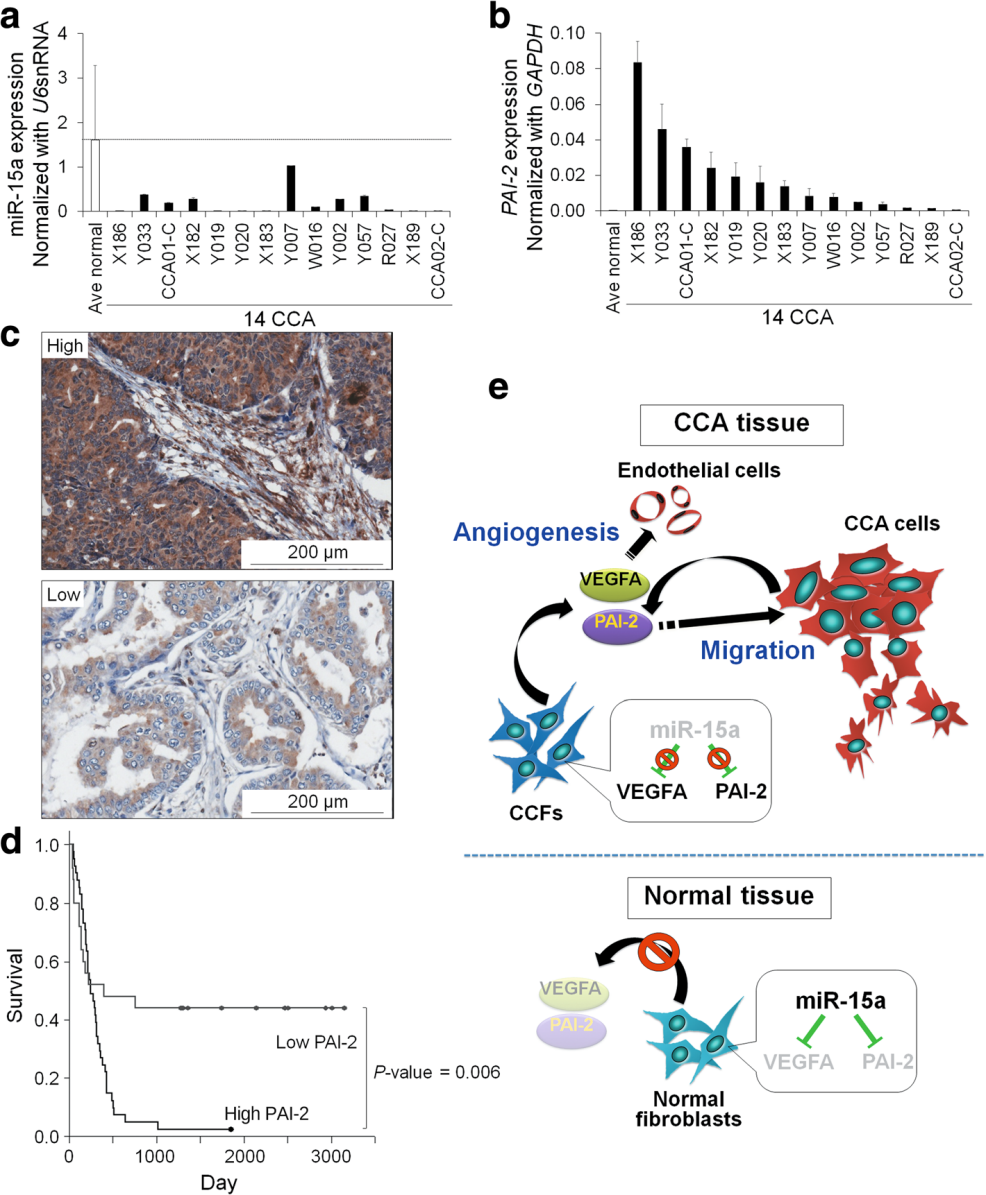
Expression of *PAI-2* in CCA tissues. **a** Real-time PCR of miR-15a in 14 CCA tissues and 2 normal liver tissues. Bars represent mean ± SD of three measurements. Dashed line represents the level of average expression in normal liver tissues. **b** Real-time PCR of *PAI-2* in 14 CCA tissues and 2 normal liver tissues. Bars represent mean ± SD of three measurements. **c** Immunohistochemical staining of PAI-2 in clinical CCA samples. Representative images of high and low expression of PAI-2 are shown. Scale bar: 200 μm. **d** The survival analysis by Kaplan-Meier test was performed using 3-y as the cut-off. **e** The proposed mechanism of miR-15a-PAI-2 axis in microenvironment of CCA tissue

In 14 CCA and 2 normal liver tissues, expressions of miR-15a and PAI-2 were examined by real-time PCR. Expression of miR-15a in CCA cases was lower than its average expression in normal liver tissues (Fig. 5a). PAI-2 expression was higher in most CCA cases compared with normal liver tissues (Fig. 5b).

Seventy-two clinical samples of CCA cases were enrolled in the immunohistochemistry experiment with the clinicopathological data including sex, age, clinical staging, histological grading, vascular invasion, lymph node (LN) metastasis and overall survival time (Table 1). The median age of patients was 59±8.2 y with a 1.4:1 male:female ratio. The average tumor size was 5 cm for the largest diameter of the mass. Around 75% of total cases were classified in late stage of the disease (stage IV) and vascular invasion and LN metastasis were found in 33% of the enrolled cases. The immunostaining revealed positive signals in tumor tissues with different degrees of expression (Fig. 5c). Approximately 63% of CCA cases (45 of 72) had high PAI-2 levels. Univariate analysis showed that the level of PAI-2 in cancer cells was not correlated with any clinicopathological parameters, but the Cox regression multivariate analysis showed significant correlations of PAI-2 independent of the other parameters (Table 1). The multivariate analysis revealed the potential of a high PAI-2 level being a risk factor in CCA patients at around 3.452-fold (*P*-value = 0.023). Moreover, high expression of PAI-2 was significantly correlated with histological type and vascular invasion. CCA patients with a high PAI-2 in cancer cells had a median survival time of 249 d compared with 395 d in those with low levels. Kaplan-Meier analysis showed a statistically significant correlation of a high PAI-2 level with short survival time (Fig. 5d, *P*-value = 0.006).

## Discussion

For decades, CAFs have been recognised for their impact in regulating the aggressive phenotypes of cancer cells in particular through the paracrine activity of secreted substances on cancer cells. In CCA in Thailand, which is commonly caused by the infection of the liver fluke, *Opisthorchis viverrini*, CAFs have been investigated for their activated phenotype by positive ASMA expression as a poor prognostic marker correlated with short patient survival time [5], recently supported by the finding that aggressive phenotype CAFs in intrahepatic CCA can be identified as a useful predictor for prognosis [18]. Using a mouse model, CCA-bearing animals were targeted with apoptosis induction specifically for CAFs within the tumor masses and the results revealed that CCA growth was attenuated, implicating CAFs in the control of cancer progression [19]. These studies, taken together, provide new evidence that CAFs can serve as potential therapeutic targets in CCA.

Most of the evidence published in the field of CAFs in CCA showed the function of paracrine substances able to activate CCA cells [8, 20, 21]. The aberrant production of secreted proteins from CAFs has been identified by us as the results of an alteration in gene expression [8]. Although epigenetic alterations and chromosomal alterations can be found associated with CAFs of different cancer types [22–24], several studies have also shown that alterations of miRNAs in CAFs are common and are involved in multiple functions in tumorigenesis and malignant progression [25–30]. In this study, changes of miRNA expression of CCA CCFs were investigated and miR-15a and miR-148a were selected as the most promising down-regulated miRNAs. The role of miR-15a in CAFs in controlling cancer growth, invasion and resistance to therapy has been identified in prostate cancer [13]. miR-15a, together with miR-16, can target several onco-products i.e. Bcl-2, Bmi-1, Wnt family members, and VEGF, IL-6 [31]. Moreover, VEGFA has been shown to be the target of miR-15a [32, 33]. This is the first study to identify PAI-2 as a target of miR-15a. Herein, elevated expression of PAI-2 was observed in CCFs, in comparison with SFs. Moreover, rPAI-2 significantly promoted the migration of CCA cells. Suppression of PAI-2 production from CCFs by transfection of miR-15a mimic attenuated the migration of CCA cells. These findings suggest that the miR-15a-PAI-2 axis in CCFs may be involved in the progression of CCA. Indeed, human CCA tissues with low expression of miR-15a showed high expression of PAI-2. Importantly, patients with high expression of PAI-2 showed poor prognosis.

Moreover, PAI-2 secreted from mast cells can up-regulate the expression of ASMA in dermal fibroblasts [34]. This is an important finding which may explain the fibrotic disorder in diseases of excessive fibrosis in the dermis. The precise mechanism by which PAI-2 activates ASMA has not been elucidated. It is proposed that PAI-2 may impact cellular activities by changing in the cell surface, including the stability of membrane receptors and extracellular ligands [34]. Hence it is tempting to speculate that PAI-2 secreted into the microenvironment of CCA may activate the expression of ASMA not only in the stromal fibroblasts but also in cancer cells. This may be one of the mechanisms by which PAI-2 can activate CCA cell migration. Another possibility is the finding in urothelial carcinoma that invasive properties were significantly correlated to the up-regulated expression of ASMA in stromal fibroblasts and loss of membranous E-cadherin and increased Snail, Slug, Zeb1 in cancer cells [35]. These altered characters are the hallmark of the ‘epithelial-to-mesenchymal’ transition phenomenon, increasing cancer cells’ ability to migrate and invade. This previous report supports our findings in CCA that ASMA positive stroma (as well as high PAI-2 in tumor microenvironment) correlate with poor prognosis.

In several types of cancer, miRNA expression profiling of CAFs has been reported [28, 36]. In head and neck cancer, miR-7, miR-196 and miR-335 are significantly up-regulated in CAFs compared with normal fibroblasts, and miR-7 is involved in cancer growth and migration [28]. In prostate cancer, the altered miRNA profile of normal control fibroblasts treated with IL-6 resembled that of CAFs [36]. In breast cancer, 11 dysregulated miRNAs were identified in CAFs: 3 up-regulated miRNAs, miR-221-5p, miR-31-3p, and miR-221-3p, and 8 down-regulated miRNAs, miR-205, miR-00b, miR-200c, miR-141, miR-101, miR-342-3p, let-7 g, and miR-26b [16]. Here, miR-15a and miR-148a were downregulated in both in vitro CCFs and clinical CCA tissues. This finding is supported by previous reports that miR-148a was downregulated in CAFs from oral cancers compared to normal fibroblasts. Investigation of miR-148a function demonstrated that overexpression of miR-148a in CAFs significantly impaired the migration and invasion of cancer cells by directly targeting WNT10B [17, 37]. In our study, miR-148a was also downregulated and WNT10B was identified as a target gene in CCFs.

The urokinase plasminogen activation (uPA) system is a crucial pathway for tumor invasion and establishment of metastasis. A systematic review evaluating the expression of uPA, urokinase plasminogen activator receptor (uPAR), PAI-1/SerpinE1 and PAI-2/SerpinB2 on primary esophageal, the gastro-esophageal junction, and gastric adenocarcinomas was reported and indicated that a high expression of uPA, uPAR and PAI-1 was associated with a higher risk of disease and poorer prognosis [38]. PAI-1 and PAI-2 are efficient endogenous inhibitors of uPA. They are key players in the breakdown of extracellular matrix and basement membrane [39]. Here, high expression of PAI-2 was observed and correlated with poor prognosis in CCA patients. PAI-2, previously detected in peripheral blood lymphocytes of CCA patients was proposed as part of a set of markers of independent and statistically significant predictors of patient survival [40]. We suggest that in addition to lymphocytes, cancer cells and CCFs may be additional sources of PAI-2 released into the microenvironment of CCA tissues. Importantly, PAI-2 can act in both autocrine and paracrine manners to activate migration of CCA cells.

Base on the association of liver fluke *Opisthorchis viverrini* infection and CCA oncogenesis, it is interesting to see up-regulated PAI-2 in CCA. PAI-2 has been reported to be related to the recruitment of M2 macrophages in the nematode infection of the intestine [41]. M2 cells are accepted for a certain time to induce the progressiveness of CCA [42]. Moreover, *Schistosoma japonicum* granulomas induced mouse liver PAI-2 expression [43]. It is tempting to speculate that *Opisthorchis viverrini* infection may stimulate PAI-2 overproduction released into the CCA microenvironment which may directly induce cancer cell migration (as reported in our study) and at the same time encourage a tumor-promoting environment by modulating infiltrating immune cells. This remains to be tested.

## Conclusions

In conclusion, this is the first report on miRNA profiling in CCFs. Downregulation of miR-15a in CCFs leads to the increased secretion of PAI-2 and VEGFA (Fig. 5e). Our findings provide the opportunity for exploring therapies aimed at reconstituting the miR-15/PAI-2 axis in CCA and the potential of using PAI-2 levels as a poor prognosis marker in these patients.

## Additional files

Additional file 1: Table S1. Primer sequences and product size. (DOCX 16 kb)

Additional file 2: Table S2. Primer sequences and product size of miRNA-targeted mRNA. (DOCX 17 kb)

Additional file 3: Figure S1. The expression of 11 cancer related genes and CAF markers that were previously reported was examined by real-time PCR in CCFs (B149 and C096) and SFs (SFA3 and SFA5). The expression level is normalized to an internal control, *GAPDH*. Bars represent mean ± SD of three measurements. (TIFF 180 kb)

Additional file 4: Figure S2. The expression levels of 15 down-regulated miRNAs in in 2 CCFs (B149 and C096) and 2 normal SFs (SFA3 and SFA5). Expression of miRNAs was examined by real-time PCR. The level is normalized to *U6 snRNA*. Graph shows the expression of miRNAs in 2 CCFs and the average level of 2 control SFs. (TIFF 194 kb)

Additional file 5: Table S3. A literature review of 15 miRNAs which are commonly down-regulated in CCFs. (PPTX 53 kb)

Additional file 6: Figure S3. The up-regulated miRNAs in CCFs. (A) Vane diagram and lists of 13 up-regulated miRNAs showing the folding compared to that in SFs. (B) Expression of up-regulated miRNAs in 2 CCFs (B149 and C096) and 2 normal SFs (SFA3 and SFA5) was examined by real-time PCR. Graph shows the expression of miRNAs in 2 CCFs and the average level of 2 control SFs. (TIFF 212 kb)

Additional file 7: Figure S4. List of candidate target genes of miR-148a. (A) Four criteria of finding the candidate target genes of miR-148a. (B) The expression levels of five predicted target genes of miR-148a including *WNT10B*, *TGFA*, *WNT1*, *TNFRSF6B* and *L-selectin* in CCFs and SFs. Scrambled miRNAs were used as a negative control. Bars represent mean ± SD of three measurements in one experiment. (C) Expression of *WNT10B* in miR-148a mimic transfected C096 CCFs was examined by real-time PCR. Bars represent mean ± SD of three measurements. *P≤0.05 compared to control. (D) Expression of *WNT10B* in miR-148a inhibitor transfected SFA3 SFs was examined by real-time PCR. Bars represent mean ± SD of three measurements. *P≤0.05 compared to control. (TIFF 200 kb)

Additional file 8: Figure S5. *VEGFA* is a target gene of miR-15a. (A) Expression of *VEGFA* in miR-15a mimic transfected C096 CCFs was examined by real-time PCR. Scrambled miRNAs were used as negative control miRNA. Bars represent mean ± SD of three measurements. (B) Expression of *VEGFA* in miR-15a inhibitor transfected SFA3 SFs was examined by real-time PCR. Bars represent mean ± SD of three measurements. (C) Expression of *VEGFA* in 6 CCFs (B149, C095, C096, D005, D012, and D017), SFs (SFA3 and SFA5) and CCA cell lines (KKU-213 and KKU-055) was examined by real-time PCR. Bars represent means ± SD of three measurements. *P≤0.05 compared to control. (TIFF 78 kb)

Additional file 9: Figure S6. The effects of rPAI-2 on CCA tumorigenic properties. (A) Cell proliferation was examined by WST assay in KKU-213 and KKU-055 at 24, 48, and 72 h after 10 μg/ml of rPAI-2 treatment. The 2% FBS containing media are used as negative controls. (B) Cell invasion by Transwell® invasion assay in KKU-213 and KKU-055. After incubation for 18 h with 10 μg/ml of rPAI-2, invaded cells were counted. Bars represent mean ± SD of three measurements. *P < 0.05 compared to control. (TIFF 4367 kb)

Additional file 10: Supplementary information of the invasion assay method. (DOCX 12 kb)

## Abbreviations

ASMA: Alpha smooth muscle actin
CAFs: Cancer-associated fibroblasts
CCA: Cholangiocarcinoma
CCFs: CCA-associated fibroblasts
miR: MicroRNA
PAI-2: Plasminogen activator inhibitor-2
SFs: Skin fibroblasts
