# Supplementary material for: The microRNA-15a-PAI-2 axis in cholangiocarcinoma-associated fibroblasts promotes migration of cancer cells
Source: Mol Cancer. 2018 Jan 18;17:10. doi: 10.1186/s12943-018-0760-x (PMC5773154; doi:10.1186/s12943-018-0760-x)
Supplement: Supplementary file 1 — Primer sequences and product size. (DOCX 16 kb) [file 12943_2018_760_MOESM1_ESM.docx]

**Additional file 1: Table S1.** Primer sequences and product size

| **Gene** | **Primer (5’to 3’)** | | | **Tm (^o^C)** | | **Size (bp)** |
| --- | --- | --- | --- | --- | --- | --- |
| ***VIM***  NM_003380.3 | | F | ATTGCAGGAGGAGATGCTTCA | | 59.44 | 151 |
|  |  | R | GTGGAGTTTCTTCAAAAAGGCA | | 57.61 |  |
| ***FAP***  NM_002423 | | F | CCAGGAGATCCACCTTTTCA | | 60.04 | 248 |
|  |  | R | TTAGCTCCAGCCTTTGGGTA | | 59.84 |  |
| ***ASMA***  NM_001613.2/NM_001141945.1 | | F | TTCAATGTCCCAGCCATGTA | | 59.92 | 222 |
|  |  | R | GAAGGAATAGCCACGGCTCAG | | 59.98 |  |
| ***FSP1***  NM_002961.2/NM_019554.2 | | F | GATGAGCAACTTGGACAGCA | | 59.99 | 110 |
|  |  | R | CTGGGAAGCCTTCAAAGAAT | | 58.38 |  |
| ***ADAM12***  NM_003474 | | F | TTTGGGGGTCAACAGTTTTC | | 59.63 | 191 |
|  |  | R | AGAGCTGGGTTCCCTTTTGT | | 59.15 |  |
| ***AREG***  NM_001657 | | F | TGGGGAAAAGCTCATGAAAA | | 55.42 | 174 |
|  |  | R | TTTCGTTCCTCAGCTTCTCC | | 57.54 |  |
| ***ER***  NM_001432 | | F | CATATGGGAGAAGGGGGAGT | | 57.88 | 166 |
|  |  | R | AAGTGCAATTACAGAGTGCAAAA | | 57.74 |  |
| ***JAGL1***  U77914 | | F | GCCTGCCTTAAGTGAGGAAA | | 57.80 | 169 |
|  |  | R | GCCAAGAACAACACATCAAAGA | | 57.69 |  |
| ***PDGF-A***  X03795 | | F | ACACGAGCAGTGTCAAGTGC | | 61.15 | 250 |
|  |  | R | TCTGGTTGGCTGCTTTAGGT | | 59.23 |  |
| ***PN***  AY140646 | | F | CACTCTTTGCTCCCACCAAT | | 58.08 | 157 |
|  |  | R | TCAAAGACTGCTCCTCCCATA | | 58.17 |  |
| ***SCG2***  NM_003469 | | F | CCCGAAGAATGATGATACCC | | 55.38 | 195 |
|  |  | R | AAATGTTGGGATTTGCTTGG | | 54.98 |  |
| ***GAPDH***  NM_002046 | | F | ACCCAGAAGACTGTGGATGG | | 59.01 | 201 |
|  |  | R | TTCTAGACGGCAGGTCAGGT | | 60.25 |  |

F: forward primer; R: reverse primer; Tm: melting temperature
