# Supplementary material for: The microRNA-15a-PAI-2 axis in cholangiocarcinoma-associated fibroblasts promotes migration of cancer cells
Source: Mol Cancer. 2018 Jan 18;17:10. doi: 10.1186/s12943-018-0760-x (PMC5773154; doi:10.1186/s12943-018-0760-x)
Supplement: Supplementary file 2 — Primer sequences and product size of miRNA-targeted mRNA. (DOCX 17 kb) [file 12943_2018_760_MOESM2_ESM.docx]

| **Gene** | **Primer** | | **Tm (^o^C)** | **Size (bp)** |
| --- | --- | --- | --- | --- |
| ***WNT1*** | F | TTCTAGACGGCAGGTCAGGT | 60.36 | 150 |
| NM_005430.3 | R | CCAACAGCAGTGGCCGAT | 60.18 |  |
| ***WNT10B*** | F | CTTCAGGGTCTGCACATCG | 60.41 | 185 |
| NM_003394.3 | R | GTGGCTACTGCGTGCATGA | 62.07 |  |
| ***TGFA*** | F | TGTGTCTGCCATTCTGGGTA | 60.11 | 182 |
| NM_003236.3 | R | TCACAGTGTTTTCGGACCTG | 59.72 |  |
| ***L-selectin*** | F | TCTGGAATCTGGTCAAATCCT | 58.58 | 153 |
| NM_000655.4 | R | CCTTGCCAGCCAAATGATAA | 60.96 |  |
| ***TNFRSF6B*** | F | CCTCAATGTGCCAGGCTCTTC | 61.29 | 130 |
| NM_003823.3 | R | GTCCTGGAAAGCCACAAAGT | 58.31 |  |
| ***FGF2*** | F | AGCGGCTGTACTGCAAAAAC | 60.45 | 163 |
| NM_002006.4 | R | GCCAGGTAACGGTTAGCACA | 61.08 |  |
| ***VEGFA*** | F | GGGCAGAATCATCACGAAGT | 60.08 | 211 |
| NM_001025366.2 | R | TGGTGATGTTGGACTCCTCA | 60.09 |  |
| ***TNFRSF13B*** | F | CTGAGTAATGAGTGGCCTGGG | 60.13 | 182 |
| NM_012452.2 | R | GGCTCTGATGGTTGCAAATGG | 60.13 |  |
| ***NRG1*** | F | CCCCGATTGAAAGAGATGAA | 60.01 | 216 |
| NM_013962.2 | R | TCCAGAATCAGCCAGTGATG | 59.79 |  |
| ***GFRA4*** | F | CCGTCACCCCTAACTACGTG | 59.83 | 143 |
| NM_145762.2 | R | CTGAATGGCACCATCCAAGC | 59.54 |  |
| ***TDGF1*** | F | CTGCCCAAGAAGTGTTCCCT | 59.89 | 161 |
| NM_001174136.1 | R | AGCATAAAAGTGGTAGTACGTGC | 59.07 |  |
| ***PAI-2*** | F | CCTGGGTCAAGACTCAAACC | 59.55 | 157 |
| NM_001143818.1 | R | CGGAAAGGATAAAGCCCATT | 60.27 |  |
| ***AXIN2*** | F | AGCCTAAAGGTCGTGTGTGG | 60.17 | 235 |
| NM_004655.3 | R | TGGCTGGTGCAAAGACATAG | 59.86 |  |
| ***FGF7*** | F | CCTGAGCGACACACAAGAAG | 59.62 | 180 |
| NM_002009.3 | R | CACAATTCCAACTGCCACTG | 60.15 |  |
| ***WNT3A*** | F | CAAGATTGGCATCCAGGAGT | 60.07 | 173 |
| NM_033131.3 | R | ATGAGCGTGTCACTGCAAAG | 60.06 |  |

**Additional file 2: Table S2.** Primer sequences and product size of miRNA-targeted mRNA

F: forward primer; R: reverse primer; Tm: melting temperature
