# Supplementary figures and images for: The microRNA-15a-PAI-2 axis in cholangiocarcinoma-associated fibroblasts promotes migration of cancer cells
Source: Mol Cancer. 2018 Jan 18;17:10. doi: 10.1186/s12943-018-0760-x (PMC5773154; doi:10.1186/s12943-018-0760-x)

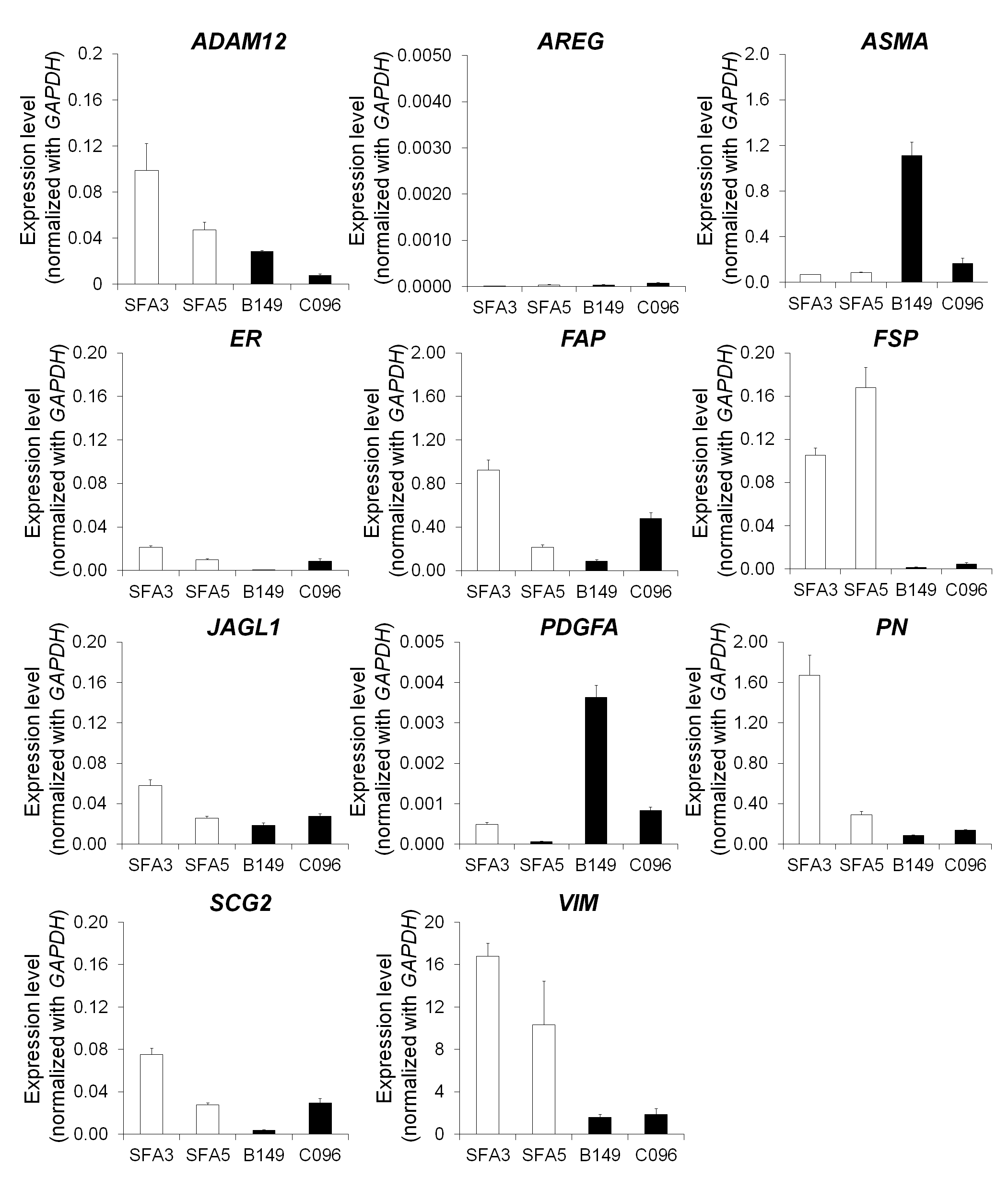

Supplement: Supplementary file 3 — The expression of 11 cancer related genes and CAF markers that were previously reported was examined by real-time PCR in CCFs (B149 and C096) and SFs (SFA3 and SFA5). The expression level is normalized to an internal control, GAPDH. Bars represent mean ± SD of three measurements. (TIFF 180 kb) [file 12943_2018_760_MOESM3_ESM.tif]

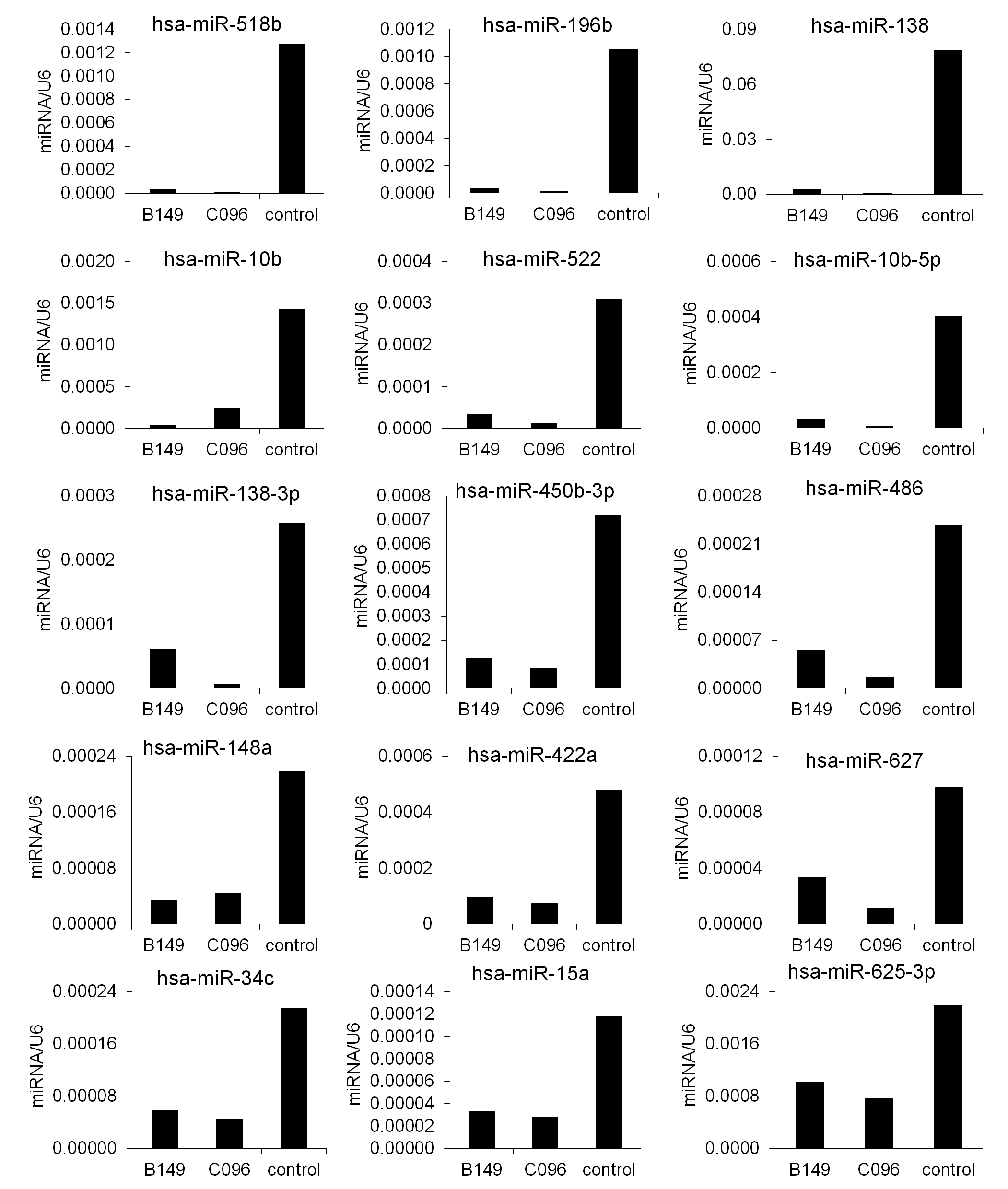

Supplement: Supplementary file 4 — The expression levels of 15 down-regulated miRNAs in in 2 CCFs (B149 and C096) and 2 normal SFs (SFA3 and SFA5). Expression of miRNAs was examined by real-time PCR. The level is normalized to U6 snRNA. Graph shows the expression of miRNAs in 2 CCFs and the average level of 2 control SFs. (TIFF 194 kb) [file 12943_2018_760_MOESM4_ESM.tif]

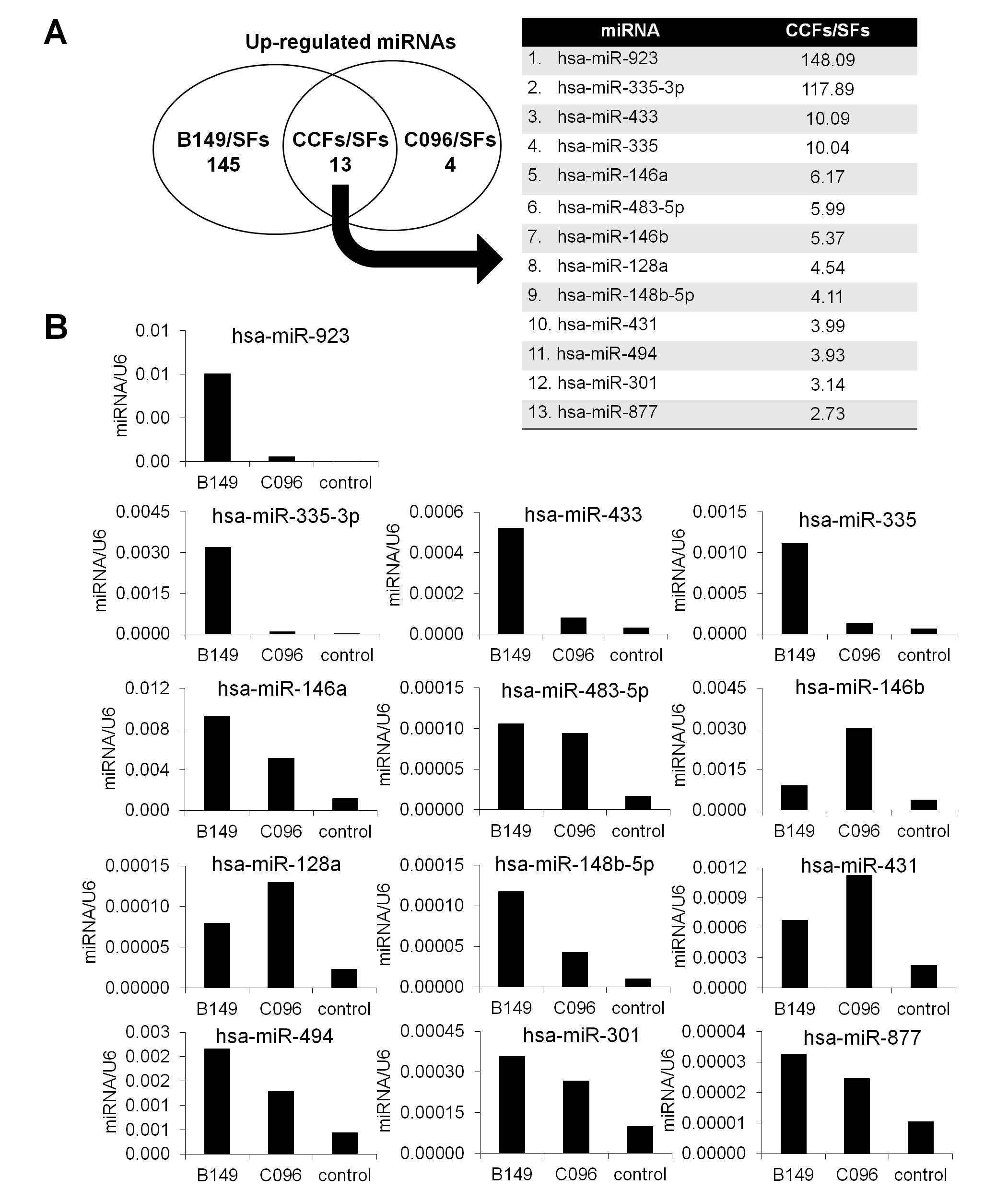

Supplement: Supplementary file 6 — The up-regulated miRNAs in CCFs. (A) Vane diagram and lists of 13 up-regulated miRNAs showing the folding compared to that in SFs. (B) Expression of up-regulated miRNAs in 2 CCFs (B149 and C096) and 2 normal SFs (SFA3 and SFA5) was examined by real-time PCR. Graph shows the expression of miRNAs in 2 CCFs and the average level of 2 control SFs. (TIFF 212 kb) [file 12943_2018_760_MOESM6_ESM.tif]

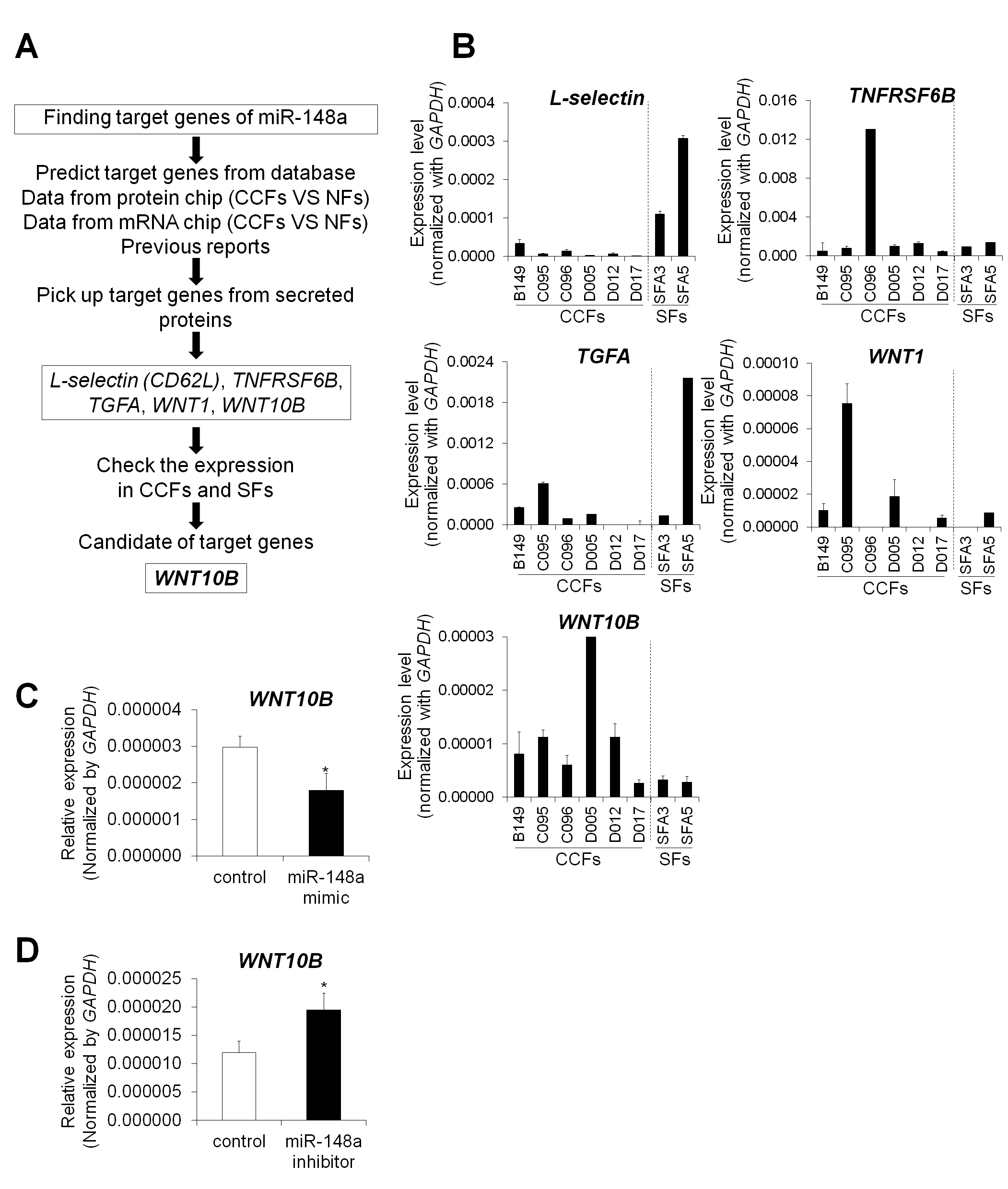

Supplement: Supplementary file 7 — List of candidate target genes of miR-148a. (A) Four criteria of finding the candidate target genes of miR-148a. (B) The expression levels of five predicted target genes of miR-148a including WNT10B, TGFA, WNT1, TNFRSF6B and L-selectin in CCFs and SFs. Scrambled miRNAs were used as a negative control. Bars represent mean ± SD of three measurements in one experiment. (C) Expression of WNT10B in miR-148a mimic transfected C096 CCFs was examined by real-time PCR. Bars represent mean ± SD of three measurements. *P≤0.05 compared to control. (D) Expression of WNT10B in miR-148a inhibitor transfected SFA3 SFs was examined by real-time PCR. Bars represent mean ± SD of three measurements. *P≤0.05 compared to control. (TIFF 200 kb) [file 12943_2018_760_MOESM7_ESM.tif]

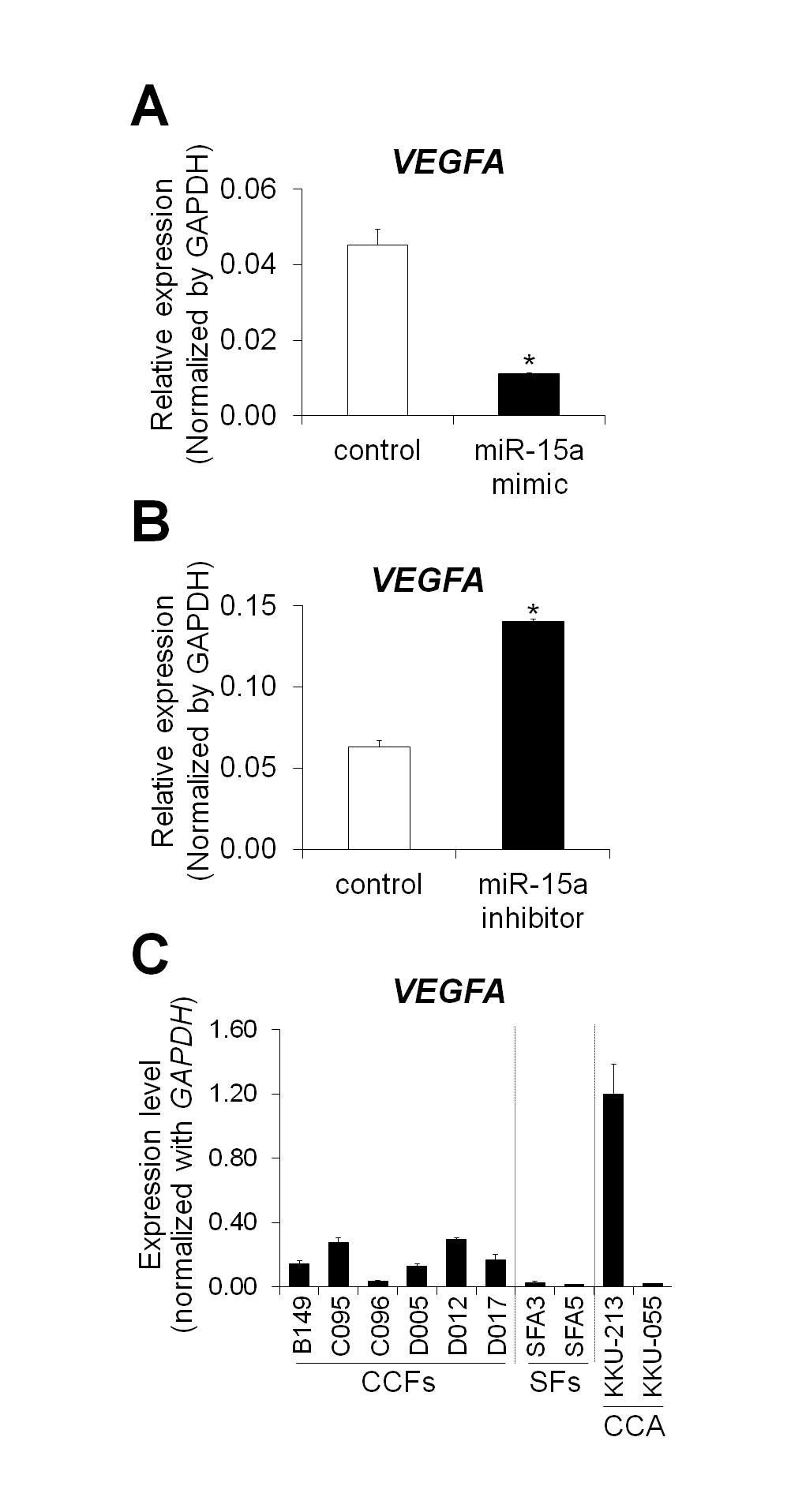

Supplement: Supplementary file 8 — VEGFA is a target gene of miR-15a. (A) Expression of VEGFA in miR-15a mimic transfected C096 CCFs was examined by real-time PCR. Scrambled miRNAs were used as negative control miRNA. Bars represent mean ± SD of three measurements. (B) Expression of VEGFA in miR-15a inhibitor transfected SFA3 SFs was examined by real-time PCR. Bars represent mean ± SD of three measurements. (C) Expression of VEGFA in 6 CCFs (B149, C095, C096, D005, D012, and D017), SFs (SFA3 and SFA5) and CCA cell lines (KKU-213 and KKU-055) was examined by real-time PCR. Bars represent means ± SD of three measurements. *P≤0.05 compared to control. (TIFF 78 kb) [file 12943_2018_760_MOESM8_ESM.tif]

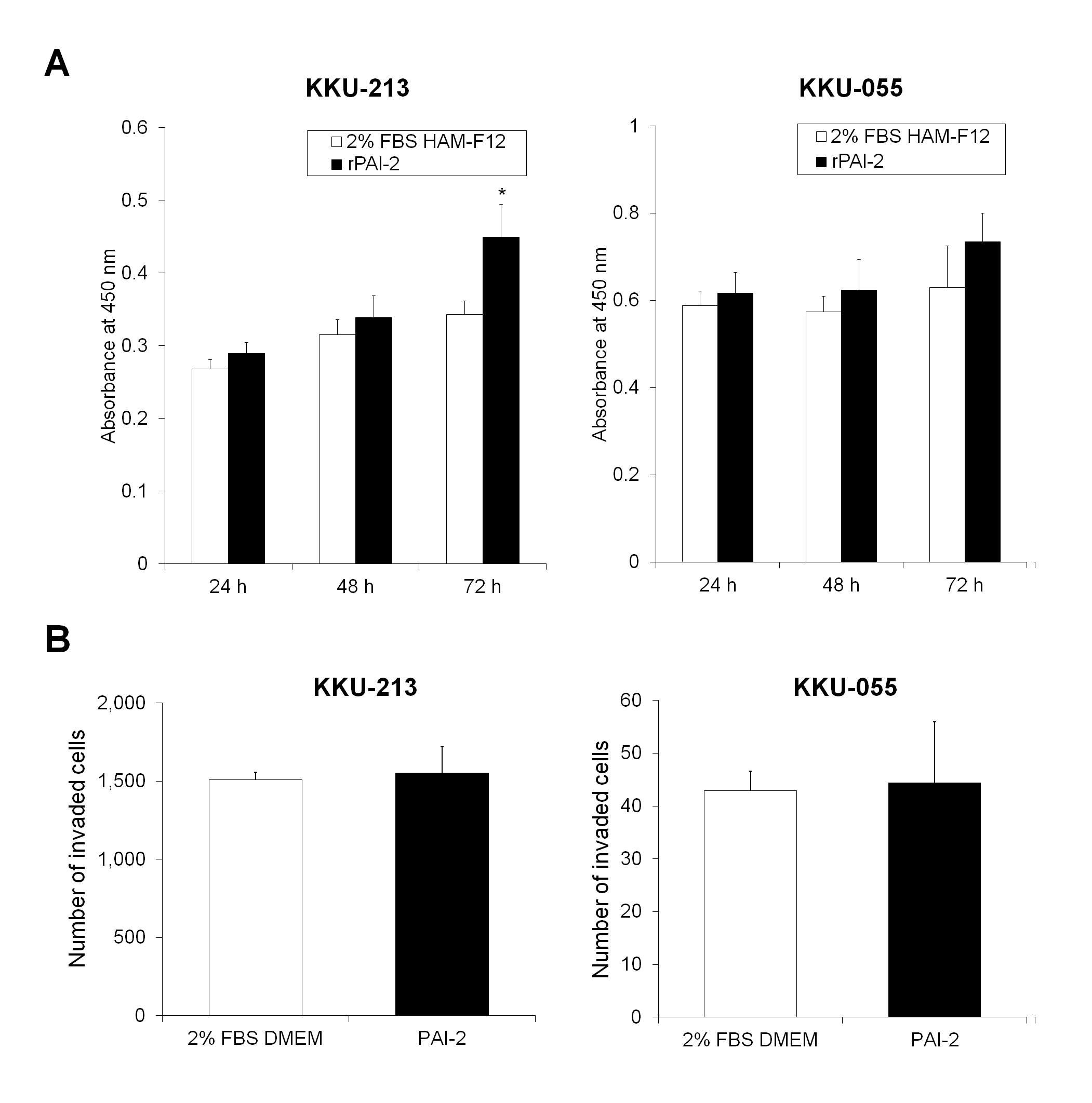

Supplement: Supplementary file 9 — The effects of rPAI-2 on CCA tumorigenic properties. (A) Cell proliferation was examined by WST assay in KKU-213 and KKU-055 at 24, 48, and 72 h after 10 μg/ml of rPAI-2 treatment. The 2% FBS containing media are used as negative controls. (B) Cell invasion by Transwell® invasion assay in KKU-213 and KKU-055. After incubation for 18 h with 10 μg/ml of rPAI-2, invaded cells were counted. Bars represent mean ± SD of three measurements. *P < 0.05 compared to control. (TIFF 4367 kb) [file 12943_2018_760_MOESM9_ESM.tif]
