# Supplementary material for: The microRNA-15a-PAI-2 axis in cholangiocarcinoma-associated fibroblasts promotes migration of cancer cells
Source: Mol Cancer. 2018 Jan 18;17:10. doi: 10.1186/s12943-018-0760-x (PMC5773154; doi:10.1186/s12943-018-0760-x)
Supplement: Supplementary file 5 — A literature review of 15 miRNAs which are commonly down-regulated in CCFs. (PPTX 53 kb) [file 12943_2018_760_MOESM5_ESM.pptx]

## Slide 1
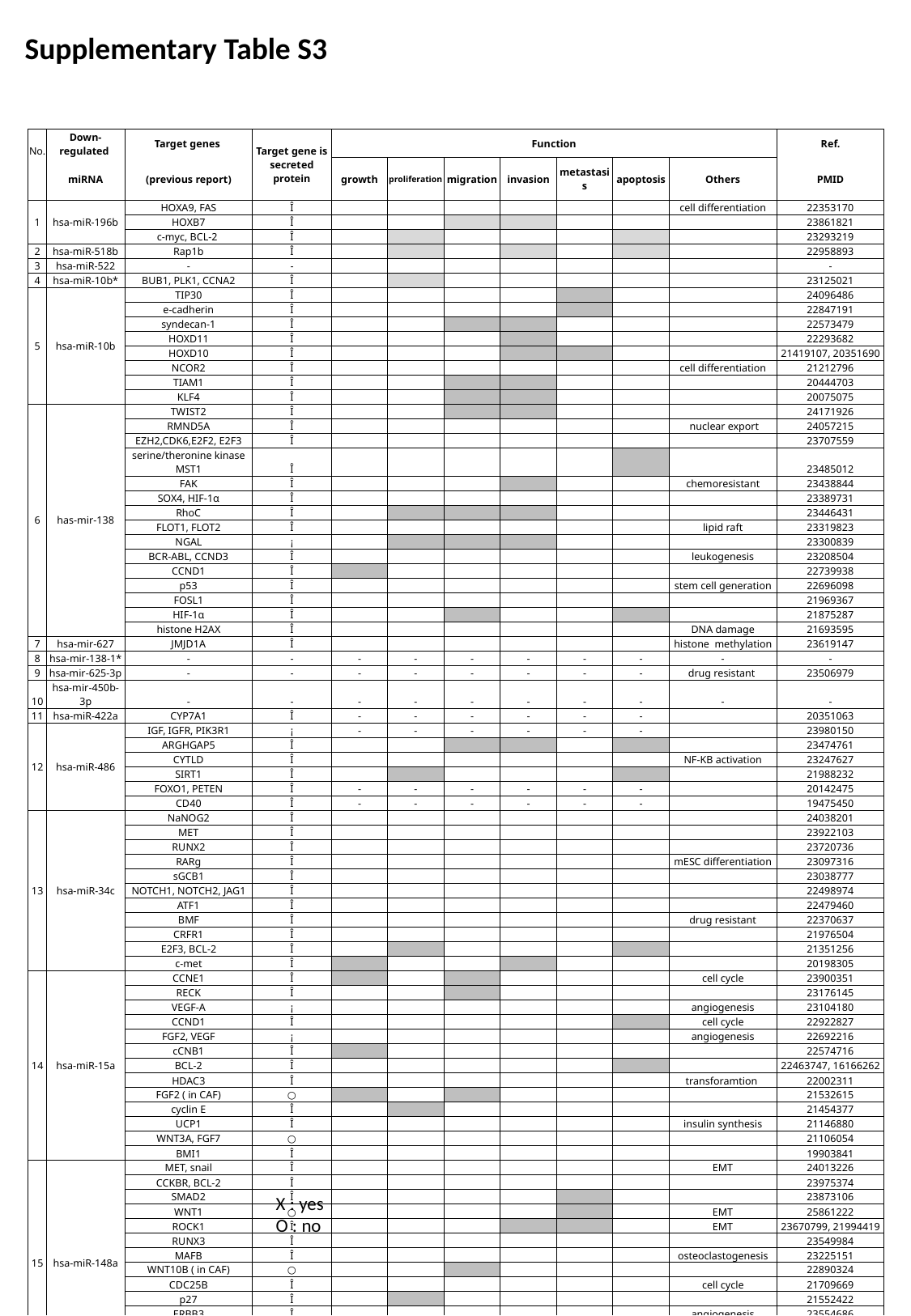

Supplementary Table S3
| No. | Down-regulated | Target genes | Target gene is secreted protein | Function | | | | | | | Ref. |
| --- | --- | --- | --- | --- | --- | --- | --- | --- | --- | --- | --- |
| | miRNA | (previous report) | | growth | proliferation | migration | invasion | metastasis | apoptosis | Others | PMID |
| 1 | hsa-miR-196b | HOXA9, FAS | Î | | | | | | | cell differentiation | 22353170 |
| | | HOXB7 | Î | | | | | | | | 23861821 |
| | | c-myc, BCL-2 | Î | | | | | | | | 23293219 |
| 2 | hsa-miR-518b | Rap1b | Î | | | | | | | | 22958893 |
| 3 | hsa-miR-522 | - | - | | | | | | | | - |
| 4 | hsa-miR-10b\* | BUB1, PLK1, CCNA2 | Î | | | | | | | | 23125021 |
| 5 | hsa-miR-10b | TIP30 | Î | | | | | | | | 24096486 |
| | | e-cadherin | Î | | | | | | | | 22847191 |
| | | syndecan-1 | Î | | | | | | | | 22573479 |
| | | HOXD11 | Î | | | | | | | | 22293682 |
| | | HOXD10 | Î | | | | | | | | 21419107, 20351690 |
| | | NCOR2 | Î | | | | | | | cell differentiation | 21212796 |
| | | TIAM1 | Î | | | | | | | | 20444703 |
| | | KLF4 | Î | | | | | | | | 20075075 |
| 6 | has-mir-138 | TWIST2 | Î | | | | | | | | 24171926 |
| | | RMND5A | Î | | | | | | | nuclear export | 24057215 |
| | | EZH2,CDK6,E2F2, E2F3 | Î | | | | | | | | 23707559 |
| | | serine/theronine kinase MST1 | Î | | | | | | | | 23485012 |
| | | FAK | Î | | | | | | | chemoresistant | 23438844 |
| | | SOX4, HIF-1α | Î | | | | | | | | 23389731 |
| | | RhoC | Î | | | | | | | | 23446431 |
| | | FLOT1, FLOT2 | Î | | | | | | | lipid raft | 23319823 |
| | | NGAL | ¡ | | | | | | | | 23300839 |
| | | BCR-ABL, CCND3 | Î | | | | | | | leukogenesis | 23208504 |
| | | CCND1 | Î | | | | | | | | 22739938 |
| | | p53 | Î | | | | | | | stem cell generation | 22696098 |
| | | FOSL1 | Î | | | | | | | | 21969367 |
| | | HIF-1α | Î | | | | | | | | 21875287 |
| | | histone H2AX | Î | | | | | | | DNA damage | 21693595 |
| 7 | hsa-mir-627 | JMJD1A | Î | | | | | | | histone methylation | 23619147 |
| 8 | hsa-mir-138-1\* | - | - | - | - | - | - | - | - | - | - |
| 9 | hsa-mir-625-3p | - | - | - | - | - | - | - | - | drug resistant | 23506979 |
| 10 | hsa-mir-450b-3p | - | - | - | - | - | - | - | - | - | - |
| 11 | hsa-miR-422a | CYP7A1 | Î | - | - | - | - | - | - | | 20351063 |
| 12 | hsa-miR-486 | IGF, IGFR, PIK3R1 | ¡ | - | - | - | - | - | - | | 23980150 |
| | | ARGHGAP5 | Î | | | | | | | | 23474761 |
| | | CYTLD | Î | | | | | | | NF-KB activation | 23247627 |
| | | SIRT1 | Î | | | | | | | | 21988232 |
| | | FOXO1, PETEN | Î | - | - | - | - | - | - | | 20142475 |
| | | CD40 | Î | - | - | - | - | - | - | | 19475450 |
| 13 | hsa-miR-34c | NaNOG2 | Î | | | | | | | | 24038201 |
| | | MET | Î | | | | | | | | 23922103 |
| | | RUNX2 | Î | | | | | | | | 23720736 |
| | | RARg | Î | | | | | | | mESC differentiation | 23097316 |
| | | sGCB1 | Î | | | | | | | | 23038777 |
| | | NOTCH1, NOTCH2, JAG1 | Î | | | | | | | | 22498974 |
| | | ATF1 | Î | | | | | | | | 22479460 |
| | | BMF | Î | | | | | | | drug resistant | 22370637 |
| | | CRFR1 | Î | | | | | | | | 21976504 |
| | | E2F3, BCL-2 | Î | | | | | | | | 21351256 |
| | | c-met | Î | | | | | | | | 20198305 |
| 14 | hsa-miR-15a | CCNE1 | Î | | | | | | | cell cycle | 23900351 |
| | | RECK | Î | | | | | | | | 23176145 |
| | | VEGF-A | ¡ | | | | | | | angiogenesis | 23104180 |
| | | CCND1 | Î | | | | | | | cell cycle | 22922827 |
| | | FGF2, VEGF | ¡ | | | | | | | angiogenesis | 22692216 |
| | | cCNB1 | Î | | | | | | | | 22574716 |
| | | BCL-2 | Î | | | | | | | | 22463747, 16166262 |
| | | HDAC3 | Î | | | | | | | transforamtion | 22002311 |
| | | FGF2 ( in CAF) | ○ | | | | | | | | 21532615 |
| | | cyclin E | Î | | | | | | | | 21454377 |
| | | UCP1 | Î | | | | | | | insulin synthesis | 21146880 |
| | | WNT3A, FGF7 | ○ | | | | | | | | 21106054 |
| | | BMI1 | Î | | | | | | | | 19903841 |
| 15 | hsa-miR-148a | MET, snail | Î | | | | | | | EMT | 24013226 |
| | | CCKBR, BCL-2 | Î | | | | | | | | 23975374 |
| | | SMAD2 | Î | | | | | | | | 23873106 |
| | | WNT1 | ○ | | | | | | | EMT | 25861222 |
| | | ROCK1 | Î | | | | | | | EMT | 23670799, 21994419 |
| | | RUNX3 | Î | | | | | | | | 23549984 |
| | | MAFB | Î | | | | | | | osteoclastogenesis | 23225151 |
| | | WNT10B ( in CAF) | ○ | | | | | | | | 22890324 |
| | | CDC25B | Î | | | | | | | cell cycle | 21709669 |
| | | p27 | Î | | | | | | | | 21552422 |
| | | ERBB3 | Î | | | | | | | angiogenesis | 23554686 |
| | | BCL-2 | Î | | | | | | | | 214555217 |
| | | CAND1 | Î | | | | | | | | 20820187 |
| | | MSK1 | Î | | | | | | | drug resistance | 20406806 |
X : yes
O : no
