# Supplementary material for: The microRNA-15a-PAI-2 axis in cholangiocarcinoma-associated fibroblasts promotes migration of cancer cells
Source: Mol Cancer. 2018 Jan 18;17:10. doi: 10.1186/s12943-018-0760-x (PMC5773154; doi:10.1186/s12943-018-0760-x)
Supplement: Supplementary file 10 — Supplementary information of the invasion assay method. (DOCX 12 kb) [file 12943_2018_760_MOESM10_ESM.docx]

**Additional file 10: Supplemental information**

**Methods**

***In vitro* invasion assay.** A 24-well cell culture insert with 8 µM pores (3097, Falcon, Becton Dickinson) was used. The membrane was coated by 20 μg of Matrigel (Becton Dickinson), which was reconstituted basement membrane substance. After trypsinization, 1.5 x 10^5^ cells were resuspended in the medium with or without 10 µg/ml of human rPAI-2 protein placed in the upper compartment of the cell culture insert for 18 h. After incubation at 37°C, the cells that penetrated the membrane into the lower side were fixed with formalin and these cells were then stained by hematoxylin. The migrated cells were determined by counting the penetrating cells onto the lower side of the filter through the pores under a microscope at x100 magnification. They were assayed 3 times and 3 fields were randomly selected and counted for each assay.
